# Mycosis fungoides progression could be regulated by microRNAs

**DOI:** 10.1371/journal.pone.0198477

**Published:** 2018-06-12

**Authors:** Rebeca Manso, Nerea Martínez-Magunacelaya, Itziar Eraña-Tomás, Verónica Monsalvez, José L. Rodríguez-Peralto, Pablo-L Ortiz-Romero, Carlos Santonja, Ion Cristóbal, Miguel A. Piris, Socorro M. Rodríguez-Pinilla

**Affiliations:** 1 Pathology Department, Fundación Jiménez Díaz, UAM, Madrid, CIBERONC, Madrid, Spain; 2 Laboratorio de Genómica del Cáncer, IDIVAL, Fundación Marques de Valdecilla, Santander, Spain; 3 Hospital de Torrejón, Pathology Department, Madrid, Spain; 4 Hospital Universitario 12 de Octubre, Dermatology Department, Madrid, Spain; 5 Hospital Universitario 12 de Octubre, Pathology Department, Madrid, CIBERONC, Madrid, Spain; 6 Translational Oncology Division, Oncohealth Institute, IIS-Fundación Jiménez Díaz, UAM, Madrid, Spain; Universitat des Saarlandes, GERMANY

## Abstract

Differentiating early mycosis fungoides (MF) from inflammatory dermatitis is a challenge. We compare the differential expression profile of early-stage MF samples and benign inflammatory dermatoses using microRNA (miRNA) arrays. 114 miRNAs were found to be dysregulated between these entities. The seven most differentially expressed miRNAs between these two conditions were further analyzed using RT-PCR in two series comprising 38 samples of early MFs and 18 samples of inflammatory dermatitis. A series of 51 paraffin-embedded samples belonging to paired stages of 16 MF patients was also analyzed. MiRNAs 26a, 222, 181a and 146a were differentially expressed between tumoral and inflammatory conditions. Two of these miRNAs (miRNA-181a and miRNA-146a) were significantly deregulated between early and advanced MF stages. Bioinformatic analysis showed FOXP3 expression to be regulated by these miRNAs. Immunohistochemistry revealed the level of FOXP3 expression to be lower in tumoral MFs than in plaque lesions in paraffin-embedded tissue. A functional study confirmed that both miRNAs diminished FOXP3 expression when overexpressed in CTCL cells. The data presented here suggest that the analysis of a restricted number of miRNAs (26a, 222, 181a and 146a) could be sufficient to differentiate tumoral from reactive conditions. Moreover, these miRNAs seem to be involved in MF progression.

## Introduction

Mycosis fungoides (MF) is the most frequent cutaneous T-cell lymphoma (CTCL) type, accounting for almost 50% of all CTCLs [1, 2] MF patients initially show cutaneous infiltration by neoplastic cells (patch, plaque and tumor), and the disease eventually progresses to include lymph node, peripheral blood or systemic involvement in late stages. Clinical and pathological diagnosis of early MF stages (patch and plaque) is difficult because of the morphological similarity to inflammatory dermatitis and the low proportion of tumoral cells [3].

MF cells have been shown to carry concurrent genetic changes in pathways regulating T-cell receptor (TCR) signaling, NFAT and NF-KB activation, and JAK/STAT signaling [4, 5]. As a result of these genetic alterations and other environmental changes or unknown factors, the phenotype of the neoplastic cells in MF may harbor markers that characterize different normal T-cell populations, including TFH (PD1, CXCL13, ICOS1, etc.), Treg (CD4, CD25, FOXP3), TH1, TH2 and TH17 cells. The clinical relevance of these changes in the phenotype has been the subject of several studies, whose results have not always been coincident [6–9].

Nevertheless, information concerning the mechanisms underlying MF genesis and progression is incomplete. MicroRNAs (miRNAs) are small non-coding RNA molecules, usually 21–22 nucleotides long, that regulate gene expression by directing mRNA degradation or repressing posttranscriptional protein translation by binding to the 3' untranslated region (UTR) of targeted gene transcripts [10–12]. They are involved in crucial biological processes, including cell growth and proliferation, differentiation and apoptosis [13, 14]. MiRNAs may have oncogenic or tumor-suppressing properties, depending on their target genes [15–18]. Studies have demonstrated miRNA-specific signatures in different types of CTCL, suggesting that they have a role in the pathogenesis of these disorders [19–27].

We analyze the differential expression profile of inflammatory dermatitis and early-stage MF samples and validate the results by analyzing two additional series of samples corresponding to early and advanced MF cases. Results are correlated with the expression level of FOXP3, a gene whose expression plays an essential role in Treg differentiation and MF progression.

## Materials and methods

### Patient samples

The series included an initial set of 14 early MF (patches-plaques) with freshly frozen samples and 15 reactive skin lesions (inflammatory dermatitis). An independent set of 65 formalin-fixed paraffin-embedded (FFPE) MF biopsies (38 plaques and 27 tumors) and 18 reactive skin lesions were used for validation experiments. No normal skin tissue was obtained. All these samples were retrieved from the cutaneous lymphoma registry of the dermatology service of Hospital Universitario 12 de Octubre (Madrid, Spain) and the Biobank of Fundación Jiménez Díaz (Madrid, Spain). All patients gave informed consent to be included in this study. Diagnoses were established according to the WHO-EORTC classification for cutaneous lymphomas [28]. Stages were defined following the EORTC scheme [29].

### Extraction of mRNA

For miRNA hybridization of fresh frozen samples, total RNA was isolated by TRIzol reagent (Invitrogen, Carlsbad, CA, USA), following the manufacturer’s instructions. RNA quality was checked using total RNA (small fraction chip) with the Agilent 2100 Bioanalyzer (Agilent Technologies Inc., Santa Clara, CA, USA), following the standard procedure.

For quantitative PCR of FFPE samples, mRNA was extracted using the RNeasy FFPE kit (Qiagen Inc., Valencia, CA, USA), in accordance with the manufacturer’s protocol.

### Microarray procedures: miRNA hybridization

Total RNA (100 ng) was hybridized on an Agilent 8x15K human miRNA microarray, following the manufacturer’s instructions (Agilent Technologies) [30]. Scanning was carried out immediately using the Agilent G2565AA Microarray Scanner System (Agilent Technologies) and data were collected with Feature Extraction v9.5 software (Agilent Technologies). Significant miRNAs (p<0.05) were represented by a cluster using Babelomics 4.2 software (http://babelomics.bioinfo.cipf.es). The microarray is available at the Gene Expression Omnibus under accession number GSE109421.

### RT quantitative PCR (qRT-PCR)

We used commercial TaqMan microRNA Expression Assays (Applied Biosystems, Foster City, CA, USA) probes against miRNA-142-3p, miRNA-146a, miRNA-186, miRNA-142-5p, miRNA-222, miRNA-181a, miRNA-502-3p and miRNA-26a. MiRNA expression of FFPE tissues was achieved using the Applied Biosystems 384-well multiplexed real-time PCR assay with 10 ng of total RNA. RNA from each case was reverse-transcribed using the TaqMan® MicroRNA Reverse Transcription kit (Applied Biosystems). miRNA-qRT-PCR was performed using TaqMan® MicroRNA Assay (Applied Biosystems). All reactions were run on the ABI PRISM HT 7900 Real-Time Sequence detection system (Applied Biosystems), in accordance with the manufacturer’s protocol. Two noncoding RNAs (RNU44 and RNU6B) were used as endogenous RNAs. Ct values were exported using Sequence Detection System version 2.2.2 software (Applied Biosystems) and the data were analyzed with Real Time StatMiner (Integromics). Reproducibility of triplicate curves was evaluated: inconsistent replicates were omitted. An miRNA was considered to be present if the Ct was less than 36 in all three biological replicates.

### MiRNA target searching

A variety of web resources and algorithms to investigate potential miRNA targets were used: miRanda (http://www.miRbase.org), miRNA miRNASVR score (http://www.microrna.org), Targetscan (http://www.targetscan.org) and PicTar (http://pictar.mdc-berlin.de/). Genes involved in any of the NF-KB, STAT/JAK or FOXP3 pathways were found.

### Statistical analysis

∆Ct values were used for statistical analysis. A limma t-test was performed (http://pomelo2.bioinfo.cnio.es) and miRNAs with associated values of p<0.05 were considered significant.

Associations between clinicopathological characteristics and expression of the significant miRNAs were assessed by Pearson correlation analysis. Estimates were considered statistically significant for values of p<0.05. All analyses were carried out with IBM SPSS Statistics v.20.0 (IBM Corp., Armonk, NY, USA).

### Immunohistochemical studies

Immunohistochemical (IHC) staining of samples was performed by the EnVision method with a heat-induced antigen-retrieval step. Sections were immersed in boiling 10 mM sodium citrate at pH 6.5 for 2 min in a pressure cooker. The expression of FOXP3 was analyzed using the antibody developed by Roncador *et al*. [31]. Intensity of staining and percentage of tumoral cells were used to score markers. Cases were considered positive when more than 10% of the tumoral cells showed nuclear immunoreactivity. The primary antibodies were omitted to provide negative controls.

### Cell cultures

HH (aggressive CTCL) was obtained from the American Type Cell Collection (ATCC, Rockville, MD, USA); My-La (mycosis fungoides) and Hut-78 (Sézary’s syndrome) were obtained from the European Collection of Cell Cultures (ECACC, Salisbury, UK).

All cell lines used are non-adherent cells and grow up in suspension. They were cultured with Roswell Park Memorial Institute 1640 (RPMI) medium, supplemented with 10% heat-inactivated fetal bovine serum (FBS) (Life Technologies), glucose (4.5 g/L), L-glutamine (292 mg/L), streptomycin sulfate (10 mg/L) and potassium penicillin (10000 U/L) (Gibco). Cell lines were grown at 37°C in a humidified sterile atmosphere of 95% air and 5% CO_2_.

### MiRNA transfection

6 x 10^6^ cells (My-La and HH) were resuspended in 100 μl OptiMEM® (Gibco) and electroporated with 40 μM pre-miRNA has-miRNA-146a (MIMAT0000449), pre-miRNA has-miRNA-181a (MIMAT0000256), and a pre-miRNA-negative control (miRNAVana miRNA mimic Negative Control #1) using AMAXA Nucleofector (Lonza). Transfected cells were cultured at 37°C within a humidified sterile atmosphere of 95% air and 5% CO_2_ for 24, 48, and 72 h.

### MiRNA isolation and qRT-PCR in cell lines

Total RNAs were extracted using TRIzol Reagent (Invitrogen) following the manufacturer’s instructions.

qRT-PCR for the targeted mature miRNA was performed using the TaqMan MicroRNA Assay kit (Applied Biosystems), following the protocol described above. Conversely, RNA was reverse-transcribed to cDNA using Transcriptor Universal cDNA Master (Roche Life Science). cDNA was amplified in a 7500 Fast Real-Time PCR System (Applied Biosystems) at 40 cycles, and using TaqMan Gene Expression Assays specific for *FOXP3* (Applied Biosystems). GAPDH was used as the internal control. Relative gene expression was calculated by the comparative cycle threshold (Ct) method.

### Western blot

Protein extracts were isolated using TRIzol Reagent (Invitrogen) following the manufacturer’s indications, denatured and separated on a 10% SDS-PAGE and western blot. Rabbit polyclonal anti-FoxP3 (Abcam) and mouse monoclonal anti-β-actin (Sigma) antibodies were used. Proteins were detected with the appropriate secondary antibodies conjugated to alkaline phosphatase (Sigma) by chemiluminescence using Tropix CSPD and Tropix Nitro Block II (Applied Biosystems).

## Results

### Comparison of mycosis fungoides with inflammatory disorders

**Fig 1:**
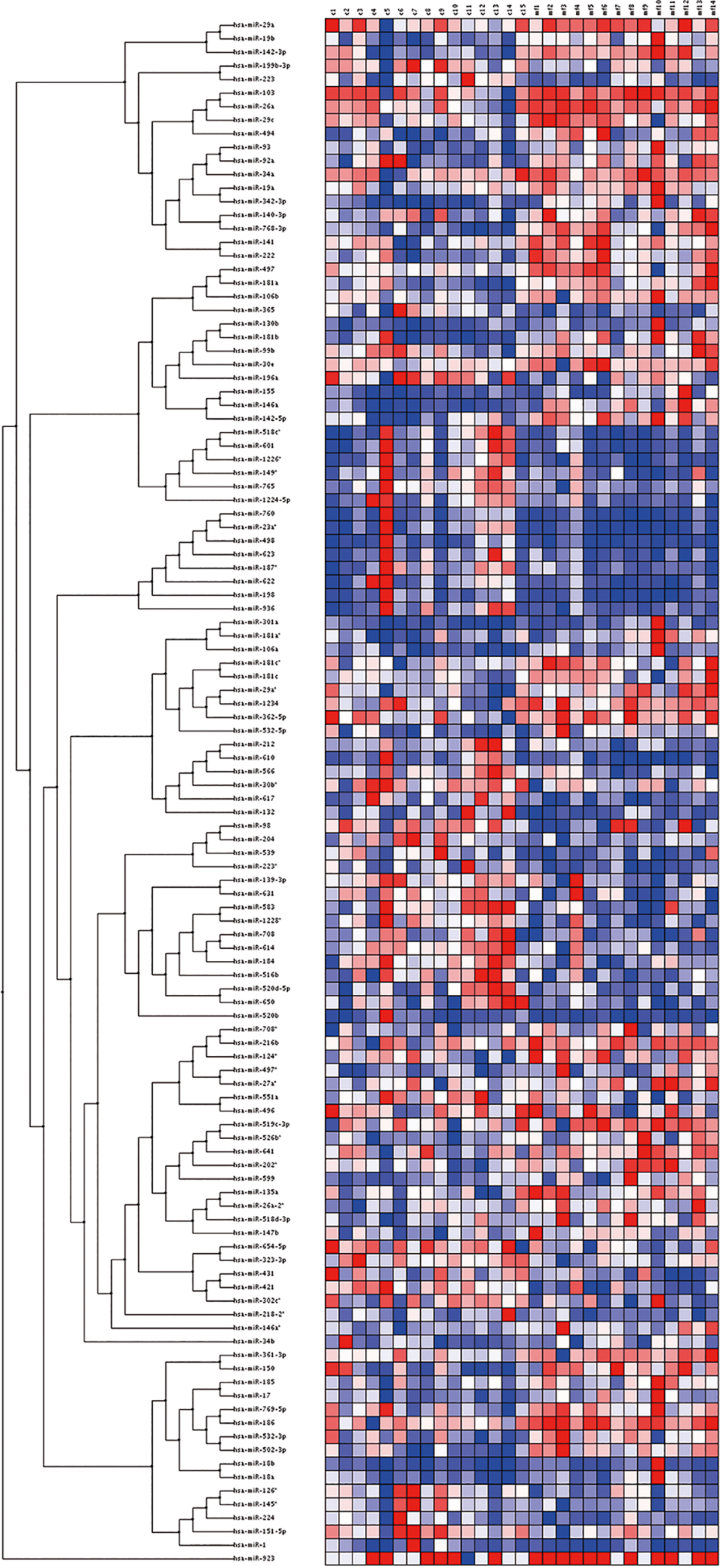
MiRNA expression heatmap of 14 MF samples and 15 skin lesions. Significant miRNAs (p<0.05) are illustrated: downregulated in blue, upregulated in red (MF: mycosis fungoides; p: probability).

We analyzed the miRNA expression profiles from the available frozen samples of 14 early MF patients and 15 inflammatory dermatitis cases using miRNA microarrays. The limma t-test is a non-permutation method used to compare expression data between two groups (http://pomelo2.iib.uam.es/help/pomelo2-help.html). Comparison of the miRNA expression profiles of MF and inflammatory dermatitis samples identified 114 differentially dysregulated miRNAs with an adjusted value of p<0.05 (Table 1) for the difference between the two conditions. Using unsupervised clustering (Fig 1), the majority of MF and control samples clustered in different groups. There were 61 upregulated miRNAs and 53 underexpressed miRNAs, respectively, in the MF group compared with the inflammatory dermatosis group.

**Table 1:** Comparison of the miRNA expression profiles of MF and inflammatory dermatitis samples identified 114 differentially dysregulated miRNAs.

Gene name	Row number	Unadj p	FDR_indep	Obs_stat	Abs(Obs_stat)
hsa-miR-142-3p	151	1.00E-05	0.0059898	-5.41705	5.41705
hsa-miR-146a	159	1.50E-05	0.0059898	-5.595763	5.595763
hsa-miR-768-3p	720	7.50E-05	0.0149744	-4.864111	4.864111
hsa-miR-186	204	7.50E-05	0.0149744	-4.169593	4.169593
hsa-miR-342-3p	374	0.0001449	0.0199659	-4.195823	4.195823
hsa-miR-142-5p	152	0.0001649	0.0199659	-4.433429	4.433429
hsa-miR-222	283	0.0001749	0.0199659	-4.42444	4.42444
hsa-miR-181a	190	0.0002399	0.0239591	-4.334453	4.334453
hsa-miR-502-3p	487	0.0003448	0.0306144	-4.351006	4.351006
hsa-miR-421	428	0.0004898	0.0391332	4.121452	4.121452
hsa-miR-26a	297	0.0006697	0.0486442	-3.800053	3.800053
hsa-miR-19b	238	0.0009046	0.0568261	-3.598278	3.598278
hsa-miR-617	650	0.0009246	0.0568261	3.364053	3.364053
hsa-miR-614	645	0.0013894	0.0783108	3.589365	3.589365
hsa-miR-223*	286	0.0015443	0.0783108	3.197657	3.197657
hsa-miR-181b	193	0.0017092	0.0783108	-3.521275	3.521275
hsa-miR-34b*	381	0.0017592	0.0783108	-3.19457	3.19457
hsa-miR-93	760	0.0017642	0.0783108	-3.323236	3.323236
hsa-miR-23a*	289	0.0020141	0.0846975	3.188748	3.188748
hsa-miR-99b*	781	0.002234	0.0892476	2.738806	2.738806
hsa-miR-1228*	103	0.0023789	0.0900281	3.374138	3.374138
hsa-miR-495	476	0.0024789	0.0900281	3.068271	3.068271
hsa-miR-150	171	0.0031636	0.1001148	-3.34764	3.34764
hsa-miR-103	81	0.0031786	0.1001148	-3.09778	3.09778
hsa-miR-323-3p	348	0.0033985	0.1001148	3.104854	3.104854
hsa-miR-106a	84	0.0034284	0.1001148	-2.854278	2.854278
hsa-miR-520b	536	0.0035084	0.1001148	2.245534	2.245534
hsa-miR-610	641	0.0038183	0.1051997	2.906091	2.906091
hsa-miR-184	201	0.0040282	0.1071719	3.154403	3.154403
hsa-miR-17	188	0.0041581	0.1071719	-2.88709	2.88709
hsa-miR-583	612	0.0047478	0.1185476	3.13578	3.13578
hsa-miR-765	716	0.0049977	0.1192083	3.020274	3.020274
hsa-miR-494	475	0.0053376	0.1202272	-3.077097	3.077097
hsa-miR-141	149	0.0055575	0.1202272	-2.971433	2.971433
hsa-miR-19a	236	0.0055675	0.1202272	-2.905121	2.905121
hsa-miR-146a*	160	0.0058773	0.1209934	-2.773144	2.773144
hsa-miR-130b	128	0.0060123	0.1209934	-2.41529	2.41529
hsa-miR-106b	86	0.0060572	0.1209934	-2.927656	2.927656
hsa-miR-198	232	0.0062572	0.1219382	2.538484	2.538484
hsa-miR-622	655	0.006502	0.1236029	2.749491	2.749491
hsa-miR-155	179	0.006652	0.1236029	-2.678914	2.678914
hsa-miR-365	390	0.0070268	0.1276004	2.8277	2.8277
hsa-miR-139-3p	145	0.0091458	0.1581789	2.802983	2.802983
hsa-miR-216b	268	0.0092208	0.1581789	-2.787215	2.787215
hsa-miR-1234	107	0.0095606	0.1581789	-2.804768	2.804768
hsa-miR-135a	135	0.0095706	0.1581789	-2.79416	2.79416
hsa-miR-361-3p	384	0.0097006	0.1581789	-2.692572	2.692572
hsa-miR-641	678	0.0113748	0.1817697	-2.718727	2.718727
hsa-miR-566	592	0.0124543	0.1878935	2.667827	2.667827
hsa-miR-760	715	0.0125243	0.1878935	2.345336	2.345336
hsa-miR-518c*	521	0.0128591	0.1878935	2.632587	2.632587
hsa-miR-650	687	0.0130441	0.1878935	2.656334	2.656334
hsa-miR-185	202	0.013169	0.1878935	-2.624582	2.624582
hsa-miR-532-5p	555	0.0134539	0.1885903	-2.607021	2.607021
hsa-miR-362-5p	387	0.0137437	0.189332	-2.619198	2.619198
hsa-miR-29a*	315	0.0145484	0.1970196	-2.620175	2.620175
hsa-miR-34a	378	0.0148432	0.1976626	-2.445948	2.445948
hsa-miR-30b*	335	0.015283	0.2001829	2.591596	2.591596
hsa-miR-132	130	0.0158478	0.2030185	2.406819	2.406819
hsa-miR-224	287	0.0160077	0.2030185	2.409084	2.409084
hsa-miR-532-3p	554	0.0168473	0.2103284	-2.567944	2.567944
hsa-miR-936	765	0.0171972	0.211393	2.515719	2.515719
hsa-miR-1	76	0.0180568	0.2175391	2.225344	2.225344
hsa-miR-223	285	0.0182417	0.2175391	2.49052	2.49052
hsa-miR-654-5p	692	0.0187165	0.2199187	2.523335	2.523335
hsa-miR-599	630	0.0203607	0.2357714	-2.396984	2.396984
hsa-miR-1226*	100	0.0216801	0.2390053	2.410134	2.410134
hsa-miR-181a*	191	0.0217701	0.2390053	-2.419391	2.419391
hsa-miR-181c*	195	0.0223798	0.2390053	-2.421737	2.421737
hsa-miR-769-5p	723	0.0224048	0.2390053	-2.440104	2.440104
hsa-miR-204	250	0.0224348	0.2390053	2.399345	2.399345
hsa-miR-145*	158	0.0233744	0.2457384	2.367751	2.367751
hsa-miR-526b*	552	0.0244089	0.2519289	-2.339147	2.339147
hsa-miR-516b	513	0.0245938	0.2519289	2.396017	2.396017
hsa-miR-202*	248	0.0249586	0.2524298	-2.383771	2.383771
hsa-miR-301a	322	0.0268578	0.2579882	-2.034438	2.034438
hsa-miR-431	437	0.0268978	0.2579882	2.345151	2.345151
hsa-miR-519c-3p	530	0.0269877	0.2579882	-2.332903	2.332903
hsa-miR-601	632	0.0270527	0.2579882	2.306771	2.306771
hsa-miR-518d-3p	522	0.0271227	0.2579882	-2.326572	2.326572
hsa-miR-923	753	0.0282621	0.264268	-2.31362	2.31362
hsa-miR-520d-5p	539	0.0292367	0.264268	2.301873	2.301873
hsa-miR-29c	319	0.0292567	0.264268	-2.307131	2.307131
hsa-miR-26a-2*	299	0.0293267	0.264268	-2.278101	2.278101
hsa-miR-551a	576	0.0294366	0.264268	2.290954	2.290954
hsa-miR-498	480	0.0300163	0.2664784	2.028779	2.028779
hsa-miR-539	556	0.0303912	0.2668412	2.267326	2.267326
hsa-miR-29a	314	0.0317356	0.2709842	-2.234032	2.234032
hsa-miR-623	656	0.0318105	0.2709842	2.196503	2.196503
hsa-miR-1224-5p	96	0.0318805	0.2709842	2.256368	2.256368
hsa-miR-98	777	0.0324952	0.2733019	2.274052	2.274052
hsa-miR-147b	164	0.03299	0.273636	-2.223571	2.223571
hsa-miR-27a*	303	0.0332199	0.273636	-2.243363	2.243363
hsa-miR-212	263	0.0345793	0.2772068	2.195928	2.195928
hsa-miR-149*	170	0.0346942	0.2772068	2.232198	2.232198
hsa-miR-187*	207	0.035249	0.2788507	2.200757	2.200757
hsa-miR-708*	711	0.0359486	0.2806747	-2.186849	2.186849
hsa-miR-126*	119	0.0364434	0.2806747	2.19159	2.19159
hsa-miR-181c	194	0.0365334	0.2806747	-2.200084	2.200084
hsa-miR-140-3p	147	0.037453	0.2849992	-2.188637	2.188637
hsa-miR-18b	212	0.0384325	0.2895797	-1.762522	1.762522
hsa-miR-218-2*	272	0.0390622	0.2895797	2.058449	2.058449
hsa-miR-631	668	0.0391422	0.2895797	2.178897	2.178897
hsa-miR-18a	210	0.041746	0.3060097	-2.034207	2.034207
hsa-miR-199b-3p	234	0.0429505	0.3100548	2.122327	2.122327
hsa-miR-124*	112	0.0434502	0.3100548	-2.12825	2.12825
hsa-miR-151-5p	174	0.0437451	0.3100548	2.114885	2.114885
hsa-miR-302c*	329	0.04385	0.3100548	2.128734	2.128734
hsa-miR-196a	228	0.0444898	0.3111929	2.118475	2.118475
hsa-miR-30e	341	0.0450595	0.3111929	-2.066863	2.066863
hsa-miR-92a	755	0.0451794	0.3111929	-2.117883	2.117883
hsa-miR-708	710	0.0459591	0.3138574	2.097904	2.097904
hsa-miR-497	478	0.0478682	0.3214009	-2.06627	2.06627
hsa-miR-497*	479	0.0495125	0.3295512	-2.031933	2.031933
hsa-miR-940	769	0.0503321	0.3295512	2.051011	2.051011
hsa-miR-483-5p	457	0.050522	0.3295512	2.029357	2.029357
hsa-miR-886-5p	738	0.0507319	0.3295512	-2.035657	2.035657
hsa-miR-205	251	0.0514716	0.3316596	-1.981135	1.981135
hsa-miR-298	311	0.0521363	0.3332551	2.028814	2.028814
hsa-miR-134	134	0.0532308	0.3346475	2.020577	2.020577
hsa-miR-29b	316	0.0533107	0.3346475	-1.988504	1.988504
hsa-miR-659	697	0.0536106	0.3346475	1.956549	1.956549
hsa-miR-143	153	0.0546851	0.3387086	1.964598	1.964598

Abs: absorbance; FDR: false discovery rate; has: Homo sapiens; indep: independent; MF: mycosis fungoides; miR: miRNA; Obs: observation; p: probability; stat: statistical; Unadj: unadjusted

*: 3p or 5p.

### Validation of miRNA expression by qRT-PCR

**Fig 2:**
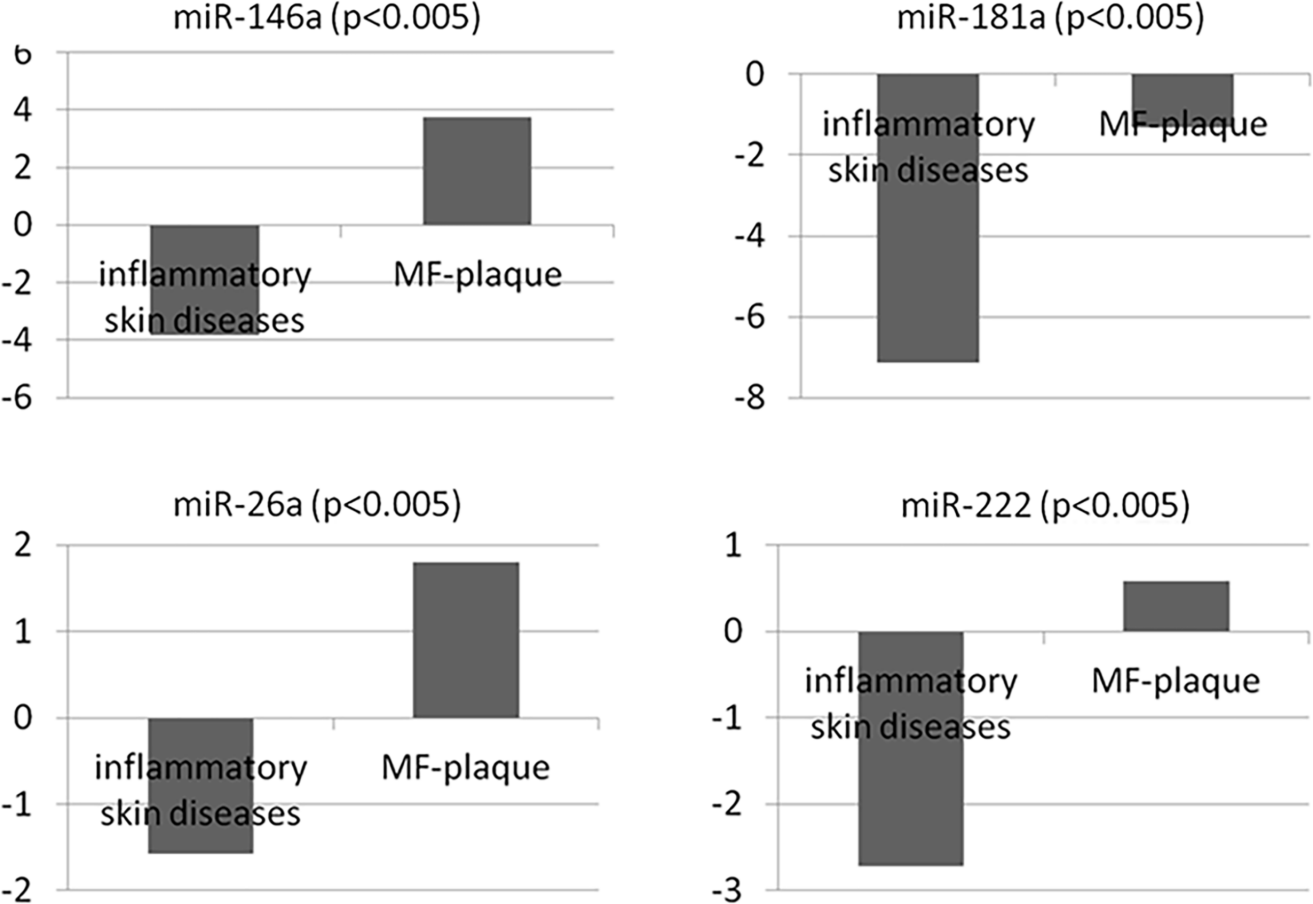
Expression levels of miRNAs in the inflammatory skin and MF FFPE samples. The -∆Ct values indicate gain of expression. These miRNAs are increasingly expressed as the disease progresses (FFPE: formalin-fixed paraffin-embedded; MF: mycosis fungoides).

Seven of the most highly upregulated miRNAs in the two groups (MF *vs*. controls) were analyzed by qRT-PCR in an independent series of 18 FFPE inflammatory dermatitis and 38 early-stage MF samples. Four miRNAs (miRNA-222, miRNA-26a, miRNA-146a and miRNA-181a) were found to be differentially expressed between the groups (Table 2). We used the -∆Ct values to represent the plots graphically and found these four miRNAs to be upregulated in the MF cases (Fig 2).

**Table 2:** miRNAs differentially expressed between the two groups (MF *vs*. controls).

Gene name	Row number	Unadj p	FDR_indep	Obs_stat	Abs(Obs_stat)
hsa-miR-26a	1	<0.0000001	<0.0000001	-9.363694	9.363694
hsa-miR-181a	3	<0.0000001	<0.0000001	-15.838742	15.838742
hsa-miR-146a	4	<0.0000001	<0.0000001	-14.477574	14.477574
hsa-miR-222	2	<0.0000001	<0.0000001	-7.029078	7.029078
hsa-miR-142-5p	5	0.1465773	0.2052082	-1.472557	1.472557
hsa-miR-502-3p	6	0.2155701	0.2514985	-1.252842	1.252842
hsa-miR-186	7	0.2593844	0.2593844	1.139632	1.139632

Abs: absorbance; FDR: false discovery rate; has: Homo sapiens; indep: independent; MF: mycosis fungoides; miR: miRNA; Obs: observation; p: probability; stat: statistical; Unadj: unadjusted.

**Fig 3:**
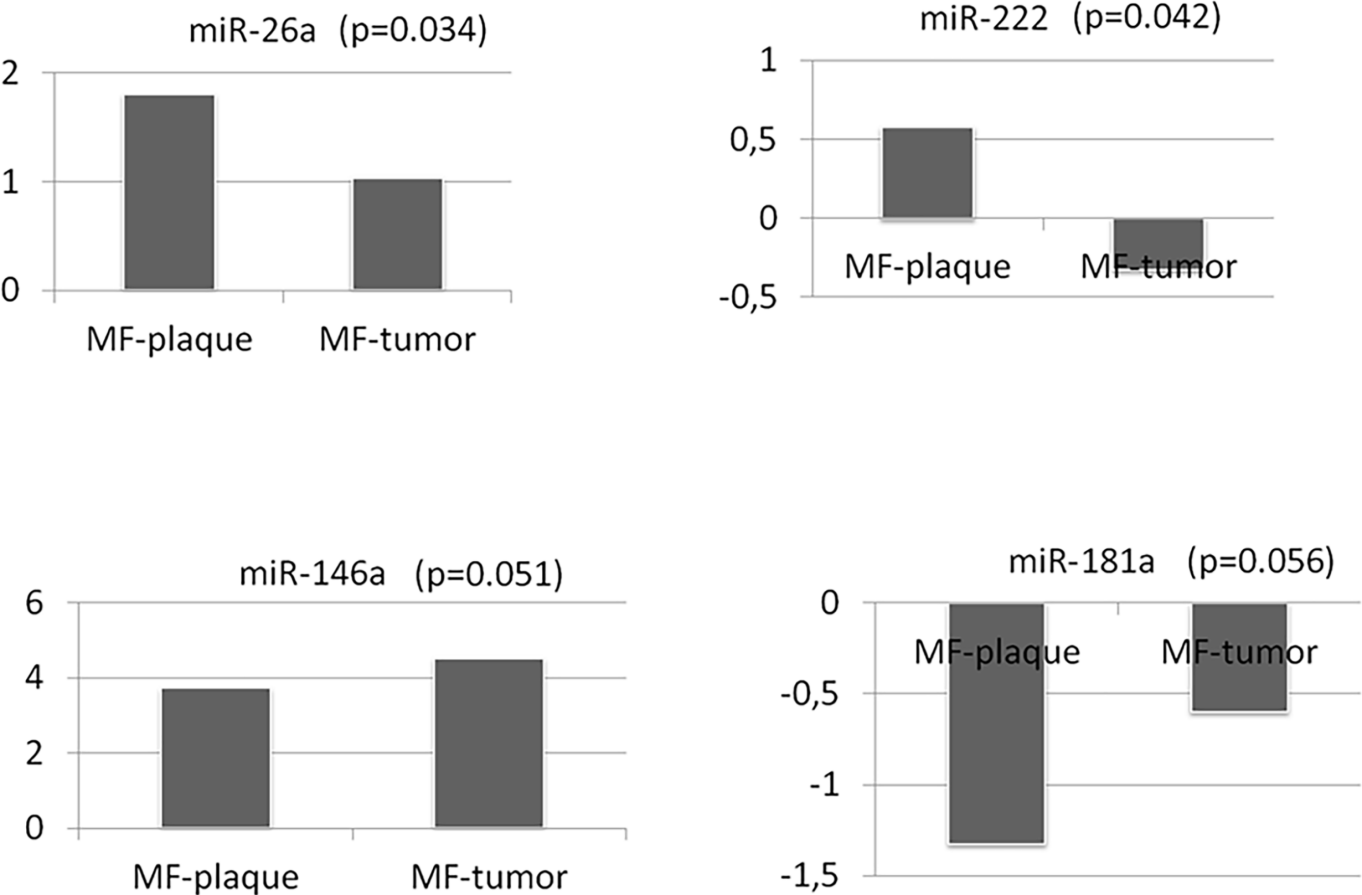
Expression levels of miRNA-26a, miRNA-222, miRNA-146a and miRNA-181a in MF FFPE samples. The -∆Ct values indicate gain of expression. These miRNAs are increasingly expressed as the disease progresses; miRNA-26a (p = 0.034), miRNA-222 (p = 0.042), miRNA-146a (p = 0.051) and miRNA-181a (p = 0.056) (FFPE: formalin-fixed paraffin-embedded; MF: mycosis fungoides).

**Fig 4:**
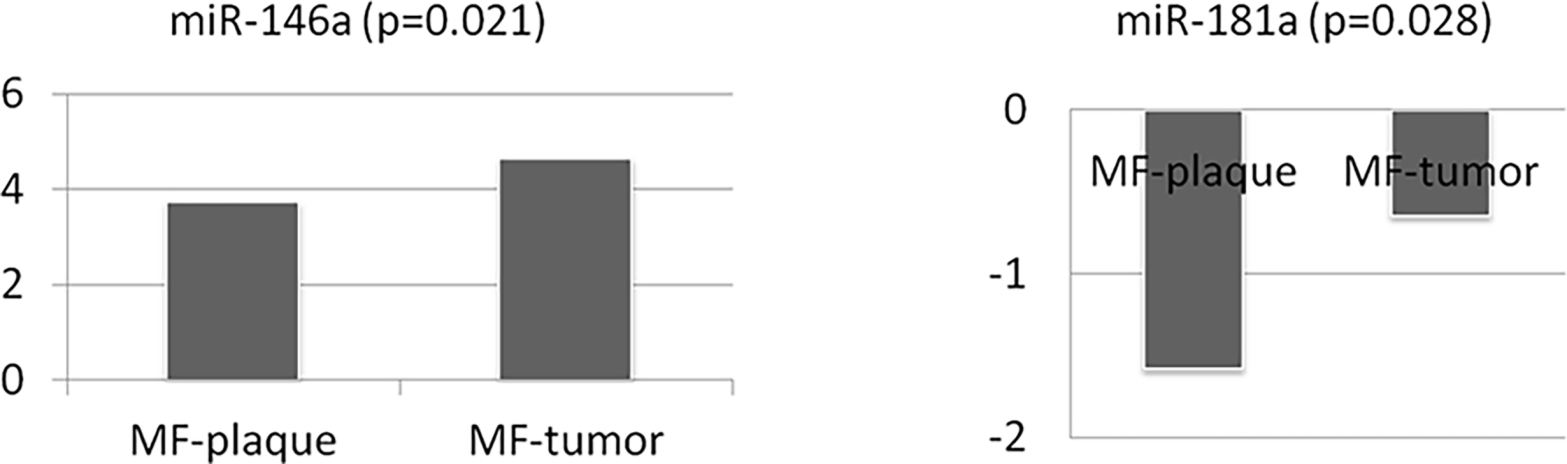
Expression levels of miRNA-146a and miRNA-181a in the MF FFPE paired samples. The -∆Ct values indicate gain of expression. These miRNAs are increasingly expressed as the disease progresses; miRNA-146a (p = 0.021) and miRNA-181a (p = 0.028). (FFPE: formalin-fixed paraffin-embedded; MF: mycosis fungoides; p: probability).

To extend the analysis, we investigated the comparative expression of the four miRNAs in samples of different MF clinical stages. To this end, a series of 38 FFPE early-stage MF and 27 advanced-stage MF samples were used. Statistical analysis revealed four differentially expressed miRNAs (Table 3); miRNA-222 and miRNA-26a were upregulated in early MF stages, while expression of miRNA-146a and miRNA-181a increased in advanced MF stages (Fig 3). When only the data from the series of 16 patients with paired samples (plaque and tumor stages) were analyzed, miRNA-146a and miRNA-181a maintained their significance (p<0.05), both being upregulated in the advanced stage MF group of tumors, as before (Table 4 and Fig 4). A positive correlation between the two miRNAs (p<0.001) was found, which suggests a possible shared regulatory mechanism.

**Table 3:** miRNAs differentially expressed between the two groups (early-stage MF vs. advanced-stage MF).

Gene name	Row number	Unadj p	FDR_indep	Obs_stat	Abs(Obs_stat)
hsa-miR-26a	7	0.0345734	0.1529545	2.151907	2.151907
hsa-miR-222	6	0.042937	0.1529545	2.058757	2.058757
hsa-miR-181a	3	0.0517351	0.1529545	-1.896134	1.896134
hsa-miR-146a	2	0.0564773	0.1529545	-1.795894	1.795894
hsa-miR-502-3p	8	0.1193114	0.1908982	1.57539	1.57539
hsa-miR-142-5p	1	0.2662212	0.3549617	-1.120028	1.120028
hsa-miR-186	4	0.9444226	0.9444226	0.069943	0.069943

Abs: absorbance; FDR: false discovery rate; has: Homo sapiens; indep: independent; MF: mycosis fungoides; miR: miRNA; Obs: observation; p: probability; stat: statistical; Unadj: unadjusted.

**Table 4:** miRNAs differentially expressed between the two groups (early-stage MF *vs*. advanced-stage MF) in paired samples.

Gene name	Row number	Unadj p	FDR_indep	Obs_stat	Abs(Obs_stat)
hsa-miR-146a	2	0.0210997	0.1127689	-2.367892	2.367892
hsa-miR-181a	3	0.0281922	0.1127689	-2.248547	2.248547
hsa-miR-142-5p	1	0.0990168	0.2004584	-1.675381	1.675381
hsa-miR-26a	7	0.1002292	0.2004584	1.669258	1.669258
hsa-miR-222	6	0.163984	0.2623744	1.408885	1.408885
hsa-miR-502-3p	8	0.2259126	0.3012168	1.223416	1.223416
hsa-miR-186	4	0.7589305	0.7589305	-0.308276	0.308276

Abs: absorbance; FDR: false discovery rate; has: Homo sapiens; indep: independent; MF: mycosis fungoides; miR: miRNA; Obs: observation; p: probability; stat: statistical; Unadj: unadjusted.

### Expression of FOXP3 in paired samples

It was possible to express FOXP3 using IHC on FFPE tissues in 11 of the 16 patients for which paired samples were available. We found positivity for FOXP3 in 81.8% (9/11) of MF-plaque samples and in 72.7% (8/11) of the MF-tumor cases analyzed. Nevertheless, there was a median of 40% positive cells in early stages compared with the 5–10% found in tumoral MF samples.

### Correlation between FOXP3 and miRNA expression in paired samples

**Fig 5:**
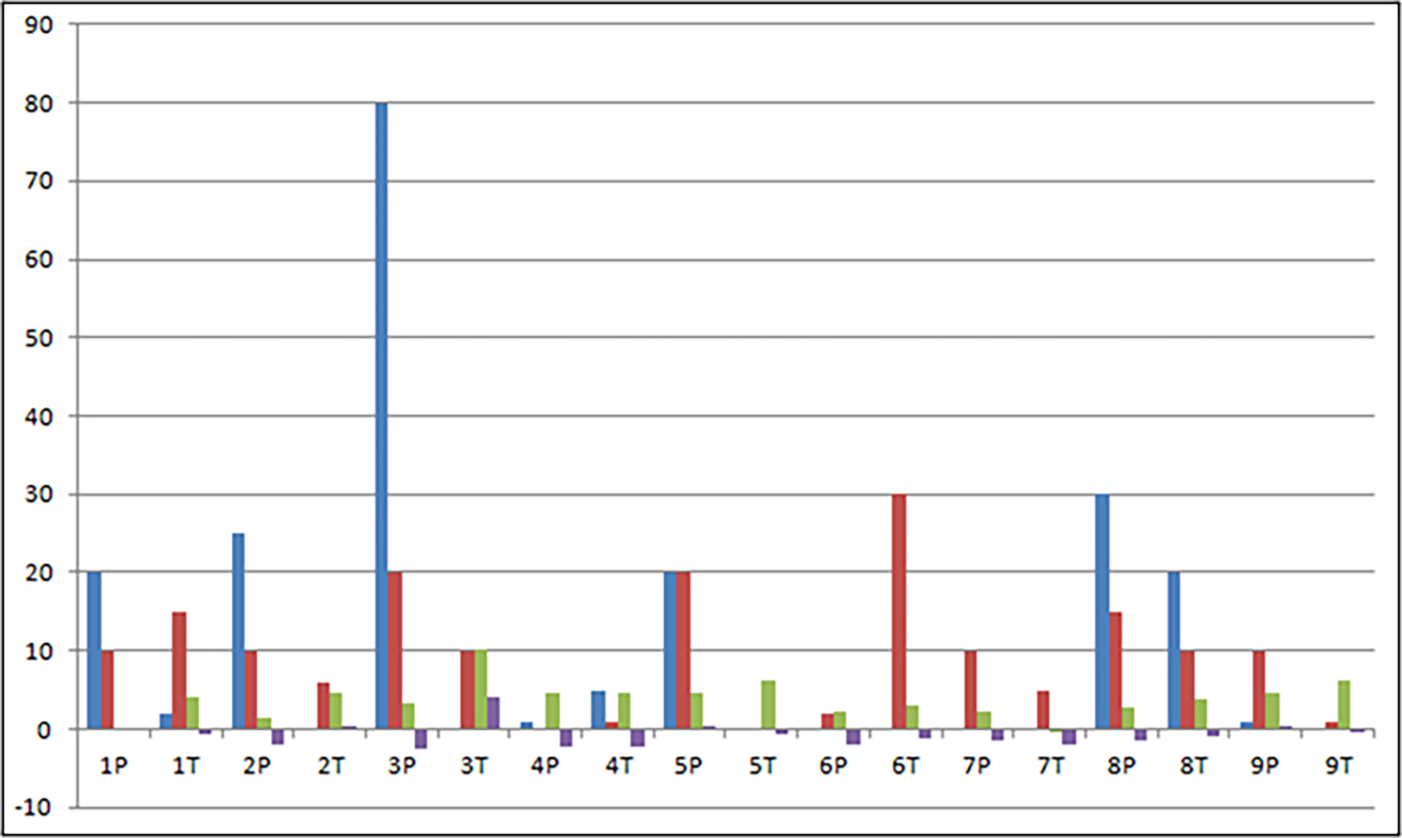
Correlation between FOXP3 and miRNA expression in paired samples. Representation of the presence and expression level, respectively, of FOXP3-positive cells and miRNAs in paired skin samples of MF patients of plaque (P) and tumoral (T) lesions. The blue bar represents intraepidermal FOXP3-positive cells. The red bar represents FOXP3-positive cells in the dermis. The green bar represents the expression level (-∆CT) of miRNA-146a. The purple bar represents the expression level (-∆CT) of miRNA-181a (MF: mycosis fungoides).

Statistical analysis showed that expression of miRNA-146a and miRNA-181a was stronger in advanced MF stages than in MF-plaque samples. FOXP3 is a target of these miRNAs [32]. We investigated whether miRNA upregulation of both miRNAs could modulate FOXP3 in paired samples of MF. As found in *in vitro* studies, we observed a reduction of FOXP3 in MF tumoral samples (Fig 5).

### Overexpression of miRNAs in MF cell lines

**Fig 6:**
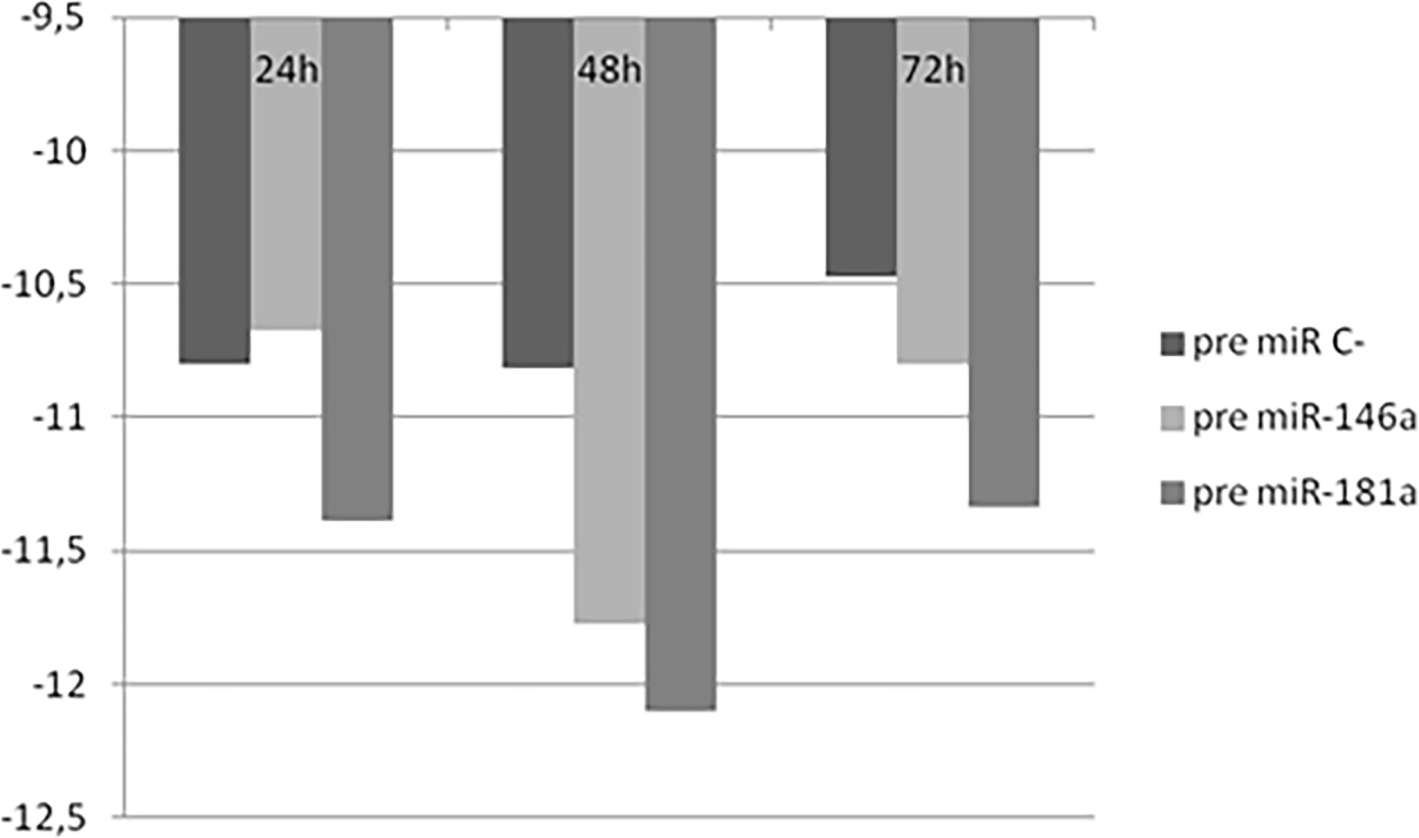
Overexpression of miRNAs in MF cell lines. A reduction in the mRNA levels of the *FOXP3* gene was found in MYLA cells with both miRNAs (MF: mycosis fungoides).

**Fig 7:**
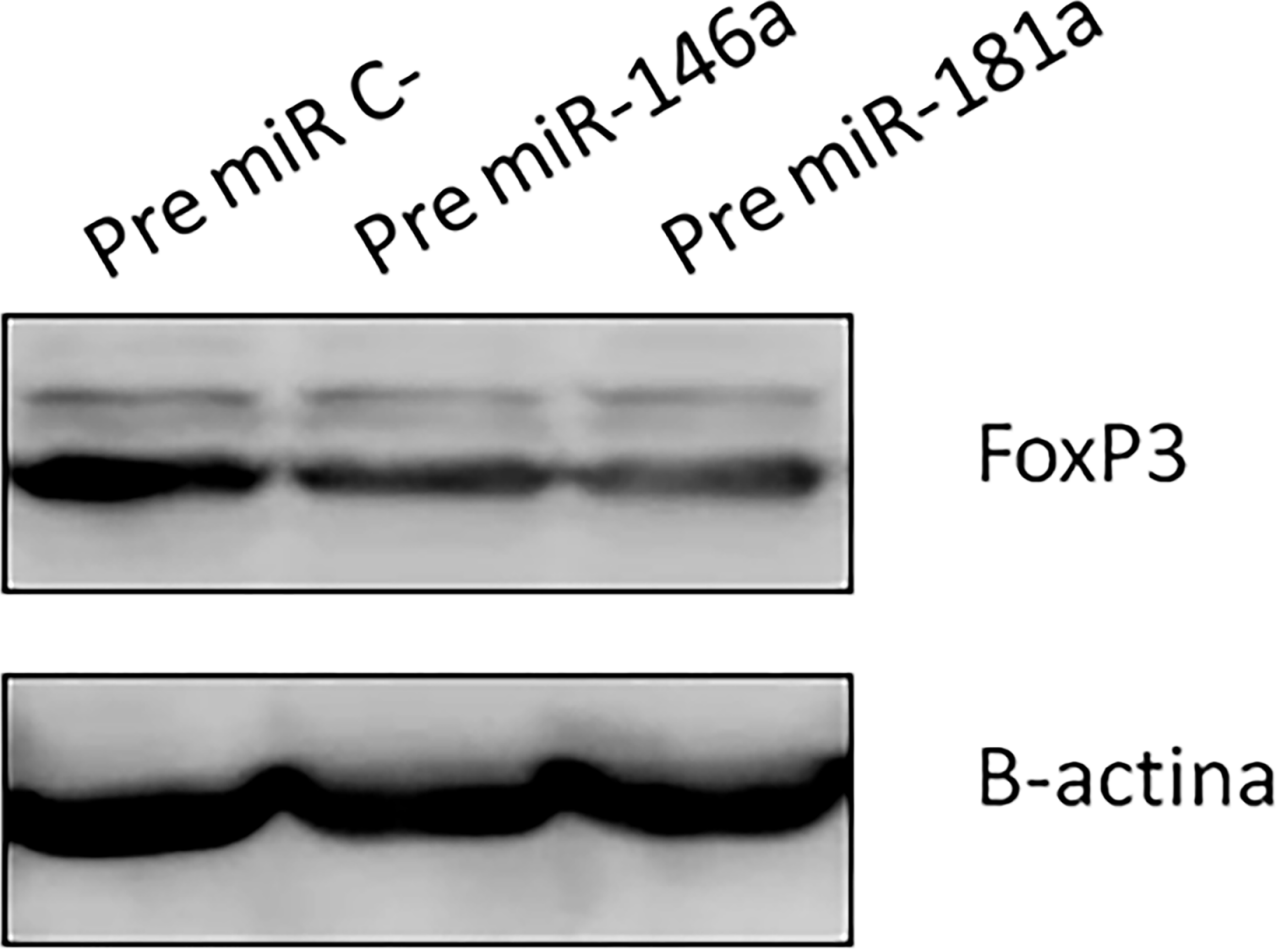
Decrease in FOXP3 protein expression levels using western blot was found again in MYLA cell line after 48 h of transfection.

We overexpressed the miRNA-146a and miRNA-181a in MYLA cells using a nucleofector and analyzed subsequent FOXP3 expression. A significant reduction in the mRNA levels of the FOXP3 gene was found in MYLA cells transfected with both miRNAs after 48 h transfection (Fig 6). Moreover, western blot revealed a decrease in FOXP3 protein expression levels in the MYLA cell line after 48 h of miRNA transfection (Fig 7).

## Discussion

This paper reports two significant strands. First, we identified a subset of miRNAs that are differentially expressed in inflammatory cutaneous conditions and early MF stages, and which could therefore be used to recognize MF. Second, we found that a more restricted set of miRNAs appears to be involved in MF progression, at least partially as a consequence of their capacity to regulate the T-cell phenotype.

MF at early stages is difficult to differentiate clinically and morphologically from other inflammatory conditions because the percentage of tumoral cells is very low. An aberrant immunophenotype (loss of CD7 expression) could be found under both neoplastic and reactive conditions [33], while clonal peaks could be found in inflammatory dermatitis as well or could not be present in early MF stages. This makes the use of other diagnostic tools essential. Quantitative real-time PCR-based miRNA profiling is a robust, reproducible technology for determining miRNA expression in paraffin-embedded tissues. We found that, in general, upregulation of an miRNA signature was associated with MF diagnosis and progression of the disease, consistent with previous reports [20–23, 26, 34]. We identified a set of 114 miRNAs that were differentially deregulated between early MF and inflammatory conditions using miRNA arrays, four of which were validated in an independent series of samples using qRT-PCR. The miRNAs of interest were miRNA-222, miRNA-26a, miRNA-146a and miRNA-181a. MiRNA-146a and miRNA-181a were also upregulated in early-stage MF relative to atopic dermatitis by Ralfkiaer *et al*. [26] and Lindahl *et al*. [34]. Our results are also consistent with those of the Van Kester *et al*. study [22], whereby most of the miRNAs we found when comparing the 19 tumoral MF and 12 dermatitis (7 lichen planus and 5 spongiotic dermatitis) cases were found to be deregulated. Furthermore, miRNA-181a was found to be upregulated in both folliculotropic MF and MF with large-cell transformation compared with inflammatory dermatosis (5 cases of chronic eczema and 3 cases of discoid lupus erythematosus) [35]. Conversely, none of them were included in the 5-miRNA classifier proposed by Ralfkiaer *et al*. [21], which was validated by Marstrand T *et al*. [36]. Ralfkiaer *et al*., using miRNA arrays, identified 27 miRNAs showing strong and highly significant differences between CTCL and both benign skin diseases and normal skin. Later on, the three most induced (miR-326, miR-663b, and miR-711) and repressed (miR-203, miR-205, and miR-718) miRNAs among the previous ones were validated in an independent series of patients using paraffin-embedded tissue and RT-PCR techniques. Furthermore, the same results were obtained from xenograft models of both CTCL and psoriasis. Ralfkiaer *et al*. [21] also validated other deregulated miRNAs previously described in the literature, such as mir-21, mir-24, mir-199, let-7b and mir-155, as being differentially expressed between tumoral and reactive conditions. Furthermore, they proposed a simplified classifier using only three of these miRNAs (mir-155, mir-205 and mir-203). These findings led them to conclude that, for diagnostic purposes, qRT-PCR was more sensitive, specific and applicable than microarrays. In our microarray study, the expression levels of miR-326, miR-663b, miR-711 and miR-203 were not significantly different from those noted in the study by Ralfkiaer *et al*. [21]. Nevertheless, mir-155 appeared to be deregulated, although we were unable to validate this. Differences between our findings and previously published data sets could be explained by the conditions required for patient selection as well as the miRNA arrays themselves. Ralfkiaer *et al*. based their first classifier 63 heterogeneous primary cutaneous T-cell lymphomas (7 anaplastic large cell lymphoma, 7 Sézary syndrome, 9 PCTCL-NOS and 39 MF, without specifying the stage of the disease) that were compared with 85 samples of both inflammatory dermatosis and normal skin [21]. To validate their first classifier they used a set of 39 nodal PTCL-NOS samples and 11 benign skin disorders. In our study we compared early-stage MF samples against inflammatory conditions, while other studies have compared all kinds of primary cutaneous T-cell lymphoma, or solely advanced MF samples. Recently, Shen X *et al*., identified another classifier comprising five miRNAs (mir-130b, mir-142-3p, mir-155, mir-200b and mir-203) which could accurately differentiate early-MF from inflammatory dermatosis [27]. Differences from the Ralfkiaer *et al*. classifier were attributed to differences in the ethnicities of the patients in both (and previous) studies.

Recently, these three miRNAs (mir-155, mir-205 and mir-203) have been identified in both CTCL tumoral cells and plasma samples in a different cohort of patients [37]. These data will allow us to correctly diagnose CTCL from benign cutaneous lesions rapidly and less aggressively, and to establish a sensitive and specific means of monitoring CTCL progression [37].

There are some characteristics that enable the miRNA data to be integrated with the morphological and phenotypic data. The presence of intraepidermal atypical T-cells, forming Pautrier’s microabscesses is a diagnostic hallmark of MF. Most TLR ligands of keratinocytes as well as CD11b of dendritic cells induce miRNA-146a production via NF-KB, leading to IL-8, TNFα and CCL20 downregulation [38–41]. Moreover, miRNA-181a directly regulates IL-8 levels by binding to the 3’ UTR region of the gene [42] and its expression is also known to be stimulated by TLR signaling [43]. Conversely, IL-8 is involved in recruiting polymorphonuclear neutrophils and it has been found not to be related to the epidermotropism of neoplastic MF T-cells [44]. Moreover, MIP-3α (CCL20) is a highly potent chemokine, predominantly expressed in extralymphoid tissue involved in chemoattraction of epithelial Langerhans-type dendritic cells and memory T-lymphocytes. It is overexpressed in atopic dermatitis, psoriasis and other cutaneous inflammatory disorders [45–47].

In general, MF is a long-duration disease that is confined to the skin, where it manifests itself as patch-plaque lesions. However, its clinical course is highly variable and, in some cases, the tumor evolves aggressively, forming cutaneous tumors and infiltrating the peripheral blood. Although many of the mechanisms involved in this process are yet to be identified, there are changes in the phenotype of the neoplastic cells associated with the progression of the disease. One of the changing markers is FoxP3, whose expression is known to decrease when comparing inflammatory dermatitis with MF, or during the progression of MF [48–50], consistent with our findings. It has already been shown that miRNA-146a and miRNA-181a could regulate FOXP3 expression directly and indirectly in a bidirectional autoregulatory loop [51–53]. We also overexpressed these two miRNAs in CTCL cell lines showing downregulation of *FOXP3* mRNA and protein expression levels. In our study, their levels of expression increased as the disease progressed.

It has been proposed that Treg differentiation is regulated by the integrated interaction of transcription factors and environmental regulation, where multiple interleukins and STAT components play an essential role [4, 5, 54, 55]. MiRNA-146a can target PRKCɛ phosphorylates STAT4 and induce its downregulation, leading to STAT3 and STAT6 overexpression [56]. STAT1 and STAT3 activation is reciprocally regulated and the components appear to play opposite roles in proliferation, apoptotic death, inflammatory and anti-tumor immune responses: STAT1 is considered to be a tumor-suppressor gene, while STAT3 is regarded as an oncogene [54]. MiRNA146a is a direct inhibitor of STAT1 transcription so its incensement as the disease progress could lead also to the overexpression of STAT3. In addition, Stat3 directly binds to the miRNA-146a promoter and induces its expression [57]. Targeting JAK/STAT-signaling pathways with chemical compounds has led to neoplastic T-cell apoptosis [5, 58–60], and inhibitors of miRNA-146a are also known to inhibit apoptosis [52, 53]. It should be noted that STAT3 is overexpressed in tumoral-stage MF specimens [60] while STAT4 downregulation is also involved in MF progression [55].

MiRNA-181a is also involved in MF progression through the direct targeting of both the PTEN and BCL2 genes and could lead to NOTCH1 overexpression by downregulating multiple negative regulators of the Notch signaling pathway, such as NRARP [61]. Notch1 is overexpressed in late stages of the disease and could be a potential therapeutic target in CTCLs [62]. Moreover, BCL2 is underexpressed in late-stage MF disease [63]. Overall, these data suggest a role for miRNA-181a in supporting proliferation and inhibiting apoptosis in MF tumoral-stage patients.

In summary, we propose that the analysis of only four miRNAs (26a, 222, 181a and 146a) could help differentiate inflammatory skin diseases from early-stage MF. These two miRNAs could also be involved in progression of the disease by controlling proliferation and apoptosis in neoplastic T-cells.
